# D2-like receptor activation does not initiate a brain docosahexaenoic acid signal in unanesthetized rats

**DOI:** 10.1186/1471-2202-15-113

**Published:** 2014-10-30

**Authors:** Ameer Y Taha, Lisa Chang, Mei Chen, Stanley I Rapoport, Epolia Ramadan

**Affiliations:** Brain Physiology and Metabolism Section, Laboratory of Neuroscience, National Institute on Aging, National Institutes of Health, Bldg. 9, Room 1S126, Bethesda, MD 20892 USA

**Keywords:** Dopamine, Quinpirole, Neurotransmission, Docosahexaenoic acid, Release, Signaling, Metabolism, Intracellular calcium, Calcium-independent phospholipase A_2_ (iPLA_2_), Calcium-dependent phospholipase A_2_ (cPLA_2_), Arachidonic acid

## Abstract

**Background:**

The polyunsaturated fatty acid, docosahexaenoic acid (DHA), participates in neurotransmission involving activation of calcium-independent phospholipase A_2_ (iPLA_2_), which is coupled to muscarinic, cholinergic and serotonergic neuroreceptors. Drug induced activation of iPLA_2_ can be measured in vivo with quantitative autoradiography using ^14^C-DHA as a probe. The present study used this approach to address whether a DHA signal is produced following dompaminergic (D)2-like receptor activation with quinpirole in rat brain. Unanesthetized rats were infused intravenously with ^14^C-DHA one minute after saline or quinpirole infusion, and serial blood samples were collected over a 20-minute period to obtain plasma. The animals were euthanized with sodium pentobarbital and their brains excised, coronally dissected and subjected to quantitative autoradiography to derive the regional incorporation coefficient, k*, a marker of DHA signaling. Plasma labeled and unlabeled unesterified DHA concentrations were measured.

**Results:**

The incorporation coefficient (k*) for DHA did not differ significantly between quinpirole-treated and control rats in any of 81 identified brain regions. Plasma labeled DHA concentration over the 20-minute collection period (input function) and unlabeled unesterified DHA concentration did not differ significantly between the two groups.

**Conclusion:**

These findings demonstrate that D2-like receptor initiated signaling does not involve DHA as a second messenger, and likely does not involve iPLA_2_ activation.

**Electronic supplementary material:**

The online version of this article (doi:10.1186/1471-2202-15-113) contains supplementary material, which is available to authorized users.

## Background

Neurotransmission underlies cognition and behavior, and when disturbed leads to changes in these functional parameters [1]. The polyunsaturated fatty acids (PUFAs), docosahexaenoic acid (22:6n-3, DHA) and arachidonic acid (20:4n-6, AA) are important second messengers in brain, where they participate in neurotransmission [2]. Agonist binding to certain neuroreceptors can release AA or DHA from the stereospecifically numbered-2 (*sn*-2) position of membrane phospholipids via activation of AA-selective group IVA calcium-dependent cytosolic phospholipase A_2_ (cPLA_2_) or DHA-selective group VIA calcium-independent phospholipase A_2_ (iPLA_2_) [3]. cPLA_2_ and iPLA_2_ are post-synaptically located [4, 5].

In the intact brain and *in vitro*, AA-preferring cPLA_2_ type IVA has been shown to be coupled to serotonergic 5-HT_2A/2C_ receptors [6, 7], cholinergic muscarinic M_1,3,5_ receptors [8–10], dopaminergic (D)2-like receptors [11–14], and ionotropic N-methyl-D-aspartate (NMDA) receptors [15, 16]. iPLA_2_, which is DHA-preferring, can be activated by agonist stimulation of cholinergic muscarinic M_1,3,5_ and serotonergic 5-HT_2A/2C_ receptors, but not of NMDA receptors [9, 16, 17].

The regulation of G-protein mediated activation of cPLA_2_ or iPLA_2_ depends on extracellular and intracellular calcium concentrations. cPLA_2_ is activated by extracellular Ca^2+^ entry into the cell and iPLA_2_ by intracellular calcium release from the endoplasmic reticulum via phospholipase C (PLC) (reviewed in [18]). PLC, when activated, converts membrane phosphatidylinositol 4,5-bisphosphate (PIP_2_) to diacylglycerol (DAG) and inositol 1,4,5-triphosphate (IP_3_). IP_3_ binding to IP_3_ receptors stimulates the release of intracellular Ca^2+^ [19, 20] and of calcium influx factor [21] from the endoplasmic/sarcoplasmic reticulum (ER/SR). The calcium influx factor contributes to the activation of iPLA_2_ by dissociating calmodulin from the active site of iPLA_2_ [22, 23]. This PLC-mediated activation of iPLA_2_ results in the hydrolysis of DHA from membrane phospholipid. DAG can also be further hydrolyzed to release DHA, although this pathway likely contributes minimally to DHA release.

Intracellular calcium release associated with activation of the PLC pathway regulates vesicle transport and neurotransmitter release at synaptic terminals [19, 24]. Muscarinic M_1,3,5_ and serotonergic 5-HT_2A/2C_ neurotransmission is coupled to iPLA_2_ via the PLC pathway, as is dopaminergic neurotransmission, although the directionality of this coupling is not agreed on. Activation of D2-like receptors in isolated neurons or dissected brain structures was reported to increase [19, 20, 25, 26] or decrease [27, 28] intracellular calcium release or IP_3_ concentration [29]. A reduction in intracellular calcium release would not activate iPLA_2_, whereas an increase would activate iPLA_2_ and increase DHA release.

In the present study, we wished to see if activation of brain D2-like receptors in unanesthetized rats would also lead to a DHA signal, as it does an AA signal [11, 12]. Stimulation of dopaminergic receptors by the agonists quinpirole or apomorphine produced an AA signal that could be blocked by the D2 receptor antagonists raclopride or butaclamol in unanesthetized rats [11, 12, 14].

To test this, we used an established *in vivo* kinetic method in awake rats [16], to quantify the DHA signal in response to the D2-like receptor agonist quinpirole, compared with vehicle. With this method, radiolabeled DHA is infused to steady state levels in plasma, and brain radioactivity is measured with quantitative autoradiography to derive the regional incorporation coefficient, k*. We found that D2-like receptor activation with quinpirole did not change the DHA incorporation coefficient (k*) into brain compared to vehicle-treated controls, suggesting that D2-like receptor activation does not involve DHA release as a second messenger.

## Methods

### Animals and diets

Experiments were conducted following the “Guide for the Care and Use of Laboratory Animals” (National Institutes of Health Publication No. 86–23) and were approved by the Animal Care and Use Committee of *Eunice Kennedy Shriver* National Institute of Child Health and Human Development. Two-month-old male Fischer CDF 344 rats (Charles River Laboratories, Wilmington, MA) were acclimated for one month in an animal facility with regulated temperature, humidity and 12-h dark/light cycle. Rats were maintained on the Rodent NIH-31 auto 18–4 diet (Zeigler Bros, Gardens, PA), which contained (as% of total fatty acid) 20.1% saturated, 22.5% monounsaturated, 47.9% linoleic, 5.1% α-linolenic, 0.02% arachidonic, 2.0% eicosapentaenoic, and 2.3% docosahexaenoic acid [30]. Water and food were provided ad libitum.

### Tracer purification and drug preparation

Radiolabeled [1-^14^C]DHA dissolved in ethanol (53 mCi/mmol, Moravek Biochemicals, Brea, CA) was purified on 60 A° thin-layer chromatography (TLC) silica plates (~5 mg per 3 cm lane on each plate) alongside phospholipid, cholesterol, cholesteryl ester, triglyceride and unesterified fatty acid standards using diethyl ether: heptane: acetic acid (60:40:3 v/v) as a solvent. The [1-^14^C]DHA was purified because the stock tracer bottles used for this study had been opened in the past, a factor which was previously found to reduce tracer purity over time despite storing it in a −80°C freezer, due to loss of the preservative argon gas blanket in the stock bottle once opened. The plate was sprayed with 0.03% 6-p-toluidine-2-naphthalene-sulfonic acid in 50 mM Tris–HCl buffer (pH7.4) (w/v), and the unesterified fatty acid band containing [1-^14^C]DHA was identified under UV light, scraped and purified from the silica particles by the Folch method (in 30 ml 2:1 v/v chloroform/methanol and 7.5 ml 0.5 M KCl). The chloroform extract was dried under nitrogen, reconstituted twice with 10 ml ethanol, centrifuged to remove additional silica particles, and pipetted to a new 50 ml Pyrex tube. The ethanol extract was reconstituted in 5 ml of ethanol. Radioactive purity measured in a portion of the ethanol extract with HPLC using acetonitrile/water (90/10%) as a solvent (constant flow rate of 2 ml/min), confirmed that 93% of the radioactivity eluted at the same time as the unesterified DHA (unlabeled) standard. On the day of the experiment, a portion of the ethanol extract was dried under nitrogen and resuspended in HEPES buffer, pH 7.4, containing 50 mg/ml fatty acid-free bovine serum albumin (Sigma-Aldrich, St Louis, MO).

An acute 1 mg/kg i.v. dose of (−)-quinpirole hydrochloride dissolved in 0.9% saline (Sigma-Aldrich) was chosen because it produces widespread significant increments in the incorporation coefficient, k*, for AA in the brain of unanesthetized rats, which can be blocked by the D2-like receptor antagonists, butaclamol or raclopride, without causing convulsions [12, 14, 31].

### Surgical procedures and tracer infusion

A total of 16 rats were randomized to saline or quinpirole treatment (n = 8 per treatment). Rats were anesthetized with halothane (2–3% v/v in O_2_) and polyethylene (PE 50) catheters were surgically inserted into the right femoral artery and vein (4 rats per day, 2 saline and 2 quinpirole) [31]. The wound was closed with surgical clips and the rat was wrapped loosely, with its upper body remaining free, in a fast-setting plaster cast taped to a wooden block. Each surgery lasted 20–25 min. Rats were allowed to recover from anesthesia for 3–4 h in an environment maintained at 25°C. Rectal temperature was maintained at 36.5-37.5°C using a feedback-heating device and rectal thermometer. Arterial blood pressure and heart rate were measured with a blood pressure recorder (CyQ 103/302; Cybersense, Nicholasville, KY). One minute after an i.v. injection of quinpirole or saline, [1-^14^C]DHA (170 μCi/kg, 2 ml) was infused into the femoral vein for 5 min at a rate of 400 μl/min, using an infusion pump (Harvard Apparatus Model 22, Natick, MA). Blood was collected at baseline and after [1-^14^C]DHA infusion (time of collection: 0, 0.2, 0.35, 0.75, 1.5, 3.0, 4.0, 4.9, 5.5, 6.5, 7.5, 10.0, and 19.0 min). Twenty min after beginning tracer infusion, the rat was euthanized with an overdose of Nembutal® (90 mg/kg, i.v.) and decapitated. The brain was removed, frozen in 2-methylbutane maintained at −40°C in dry ice, and stored at −80°C until sectioned.

### Chemical analysis

Blood samples, collected before, during or after [1-^14^C]DHA infusion, were centrifuged immediately at 18,000 *g* for 30 s at room temperature. Total lipids were extracted from plasma (30 μl) using a modified Folch procedure [32]. One hundred μl of the lower organic phase was used to determine the radiolabeled unesterified plasma [1-^14^C]DHA concentration by liquid scintillation counting.

Concentrations of unlabeled, unesterified fatty acids were determined from 100 μl of ice-thawed arterial plasma collected at 20 minutes. Unesterified heptadecanoic acid (17:0) was added as an internal standard to the plasma and total lipids were extracted with the Folch method. The total lipid extract was separated by thin layer chromatography on 60 A° silica gel plates (Whatman, Clifton, NJ) alongside phospholipid, unesterified fatty acids, triglyceride and cholesteryl ester standards using the solvent system heptane: diethylether:glacial acetic acid (60:40:3, v/v/v). The plates were sprayed with 0.03% 6-p-toluidine-2-naphthalene-sulfonic acid in 50 mM Tris–HCl buffer (pH7.4) (w/v), and the unesterified fatty acids band was identified under UV light, scraped and methylated with 1% H_2_SO_4_ (by vol) in anhydrous methanol (3 h at 70°C) after adding 0.2 ml toluene. The fatty acid methyl esters were extracted with 3 ml heptane after terminating the reaction with 1.5 ml water [33], reconstituted in 25 μl of isooctane and quantified by gas chromatography as described [30]. The injection volume was 2 μl.

### Quantitative autoradiography

Quantitative autoradiography was performed on a total of 81 brain regions from autoradiographs of coronal brain sections. The regions were identified from a stereotaxic rat brain atlas [34], and were sampled in both hemispheres. The average of bilateral measurements for each region from three consecutive brain sections was used to calculate regional radioactivity (nCi/g wet brain) by digital quantitative densitometry, using the public domain 1.62 Analysis NIH Image program. Regional brain incorporation coefficient k* (ml plasma/s/g wet brain) of [1-^14^C]DHA were calculated as follows [35]:
1

where  (nCi/g wet brain wt) is brain radioactivity 20 min after infusion,  (nCi/ml plasma) is arterial unesterified [1-^14^C]DHA concentration, and *t* (min) is time after beginning [1-^14^C]DHA infusion. Integrated plasma radioactivity (integral of c*_plasma_.dt), which corresponds to the input function, was determined by trapezoidal integration and used to calculate k*.

The regional rate of incorporation of unesterified DHA from plasma into brain phospholipids, *J*_in_ (nmol/s/g), was calculated as follows [30, 36, 37]:
2

where c_plasma_ is the plasma concentration (nmol/ml) of unlabeled unesterified DHA. Since negligible amounts of DHA (<1%) can be synthesized *de novo* by the brain from its precursor, alpha-linolenic acid [30, 36], *J*_*in*_ represents the metabolic loss of DHA by the brain [35].

### Statistical analysis

All data are presented as mean ± SD. An unpaired t-test was used to assess significant changes in body weight, baseline body temperature, arterial blood pressure and heart rate, input function, the incorporation coefficient (k*) and the incorporation rate, *J*_*in*_. A paired t-test was applied to compare mean body temperature, blood pressure and heart rate in the same animal before and after saline or quinpirole injection. Statistical significance was accepted at p < 0.05.

## Results

### Physiological parameters

One control rat died after surgery and prior to infusion with saline due to unknown causes. During the brain slicing, one control and one quinpirole brains were not sectioned uniformly, resulting in a saturated, unquantifiable signal, and a sample size of 6 saline and 7 quinpirole. Physiological parameters, plasma input function and fatty acid concentrations were therefore obtained from 6 saline and 7 quinpirole treated rats.

Table 1 shows bodyweight, body temperature, arterial blood pressure, heart rate and orofacial activity, before and after saline or quinpirole treatment. Body weight did not differ significantly between the groups. Baseline body temperature was within physiological range (36.5-37.5°C) for both groups. Baseline body temperature, heart rate and systolic and diastolic blood pressure did not differ significantly (P > 0.05 by unpaired t-test).

**Table 1:** **Physiological parameters before and after saline or quinpirole administration to unanesthetized rats**

	Saline (n = 6)		Quinpirole (n = 7)	
	Before	After	Before	After
Body weight (g)	336 ± 22		331 ± 13	
Rectal temperature (°C)	36.5 ± 1.0	37.1 ± 0.2	37.2 ± 0.3	36.6 ± 0.1**
Heart rate (beats/min)	434 ± 36	393 ± 50	441 ± 22	444 ± 20
Arterial blood pressure (mm Hg)				
Systolic	182 ± 19	164 ± 33	182 ± 7	178 ± 13
Diastolic	102 ± 13	88 ± 25	104 ± 6	95 ± 14
Number of rats with orofacial activity^1^	0/6	0/6	0/7	7/7

^1^Orofacial activity, consisting of head tremors and sniffing was observed for 10 minutes after saline or quinpirole injection. All quinpirole-treated rats exhibited characteristic head tremors and sniffing activity within 2 minutes of drug injection. Values are mean ± SD of n = 6 saline and 7 quinpirole treated rats per group. **P < 0.01 compared to before quinpirole injection by paired t-test.

Heart rate and systolic and diastolic blood pressures did not significantly change after saline or quinpirole injection, compared to baseline (P > 0.05 by paired t-test). Body temperature did not change also after saline injection, but was significantly reduced by 0.6°C after quinpirole injection (P < 0.01 by paired t-test; Table 1).

All quinpirole-treated rats but not controls exhibited orofacial head- and sniffing activity between 2 to 10 minutes after quinpirole injection, as reported [16]. No convulsions were observed in either group.

### Plasma input function

**Figure 1:**
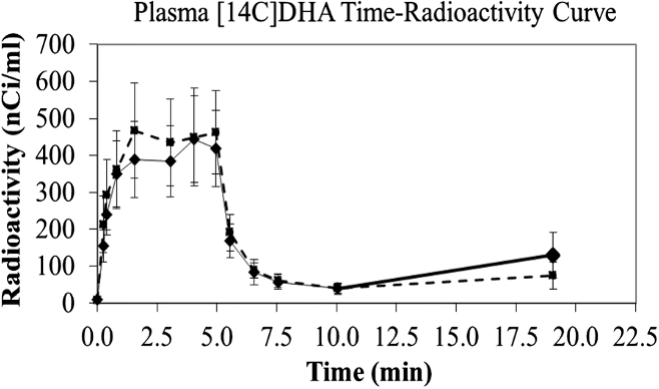
^**14**^ **C-DHA plasma radioactivity over 20 minutes in rats administered acute 1 mg/kg i.v. dose of (−)-quinpirole hydrochloride dissolved in 0.9% saline or vehicle consisting of 0.9% saline.** Solid line represents saline treated rats; dashed line represents quinpirole treated rats. Values are mean ± SD of n = 6 saline and 7 quinpirole treated rats per group.

The plasma [1-^14^C]DHA-time curve is shown in Figure 1. Mean integral radioactivity over time, which represents the input function, did not differ significantly between saline and quinpirole treated rats (quinpirole, 183834 ± 51257 nCi/ml/s, n = 7 versus saline, 184872 ± 49596 nCi/ml/s, n = 6), consistent with our previous report [38].

### Plasma unesterified fatty acid concentrations

Plasma unesterified fatty acid concentrations, including DHA concentration, did not differ significantly between the groups (Additional file 1: Table S1). DHA concentration was 13 ± 2 nmol/ml (n = 6) in controls, and 17 ± 4 nmol/ml (n = 7) in quinpirole treated rats (p = 0.096).

### Incorporation coefficient (k*)

**Figure 2:**
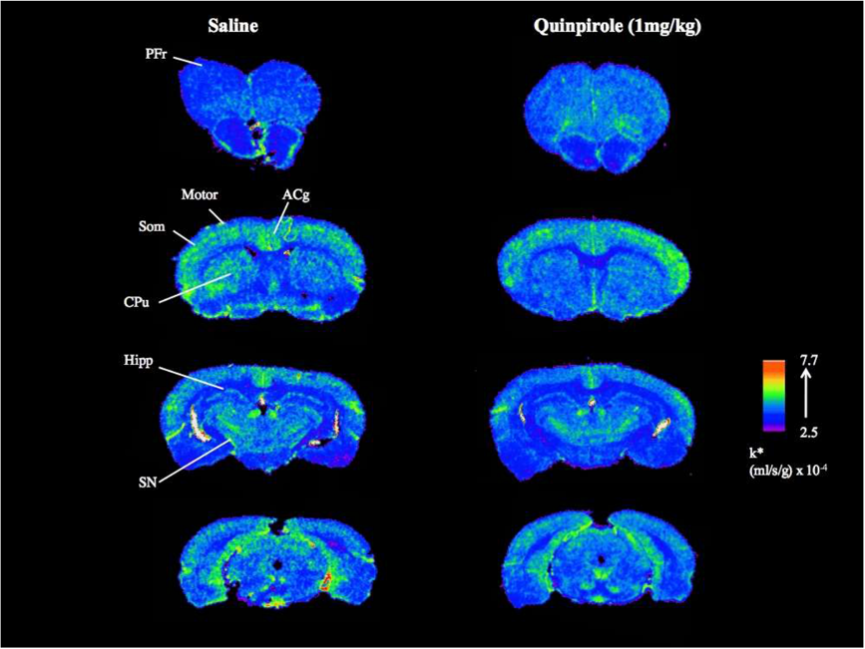
**Representative autoradiogram of coronal brain slices showing** ^**14**^ **C-DHA radioactivity following acute vehicle consisting of 0.9% saline or 1 mg/kg i.v. dose of (−)-quinpirole hydrochloride dissolved in 0.9% saline.**

Mean values of DHA incorporation coefficient k* in acute saline- and quinpirole- treated rats are presented in Table 2. Regional k* values ranged between 3.4 to 15.9 ml/s/g × 10^−4^ in saline-treated control rats, consistent with our previous report [16]. There was no significant difference in k* between quinpirole-treated and control rats in any of the 81 brain regions examined. A representative autoradiogram comparing the DHA signal following saline or quinpirole is shown in Figure 2.

**Table 2:** **Mean values of [1-** ^**14**^ C] docosahexaenoic acid incorporation coefficient k*in acute saline- and quinpirole-treated rats

Brain region	Saline (n = 6)	Quinpirole (n = 7)
***Telencephalon***		
Prefrontal cortex layer I	3.42 ± 0.87	3.00 ± 0.73
Prefrontal cortex layer IV	3.76 ± 0.98	3.66 ± 1.21
Primary olfactory cortex	3.28 ± 0.85	3.77 ± 1.76
Frontal cortex (10)		
Layer I	3.13 ± 0.79	3.88 ± 1.54
Layer IV	3.51 ± 1.00	4.26 ± 1.67
Frontal cortex (8)		
Layer I	3.38 ± 0.91	3.76 ± 1.77
Layer IV	3.66 ± 1.02	4.13 ± 1.91
Pyriform cortex	3.29 ± 0.60	3.12 ± 0.92
Anterior cingulate cortex	4.18 ± 0.93	5.06 ± 2.80
Motor cortex		
Layer I	3.35 ± 0.71	3.60 ± 1.39
Layer II – III	3.72 ± 0.88	4.39 ± 1.74
Layer IV	4.00 ± 0.92	4.42 ± 1.59
Layer V	3.35 ± 0.79	3.64 ± 1.35
Layer VI	3.25 ± 0.61	3.50 ± 1.29
Somatosensory cortex		
Layer I	3.61 ± 0.83	4.01 ± 1.57
Layer II–III	3.97 ± 0.88	4.68 ± 1.92
Layer IV	4.20 ± 1.03	4.57 ± 1.73
Layer V	4.09 ± 0.90	4.51 ± 1.72
Layer VI	3.93 ± 0.89	4.44 ± 1.72
Auditory cortex		
Layer I	4.09 ± 1.36	4.51 ± 1.29
Layer IV	4.07 ± 1.23	4.11 ± 1.22
Layer VI	3.62 ± 1.21	3.82 ± 1.18
Visual cortex		
Layer I	3.44 ± 1.10	4.17 ± 1.37
Layer IV	3.92 ± 1.24	4.52 ± 1.35
Layer VI	3.77 ± 1.14	4.38 ± 1.30
Preoptic area (LPO/MPO)	3.39 ± 0.76	3.48 ± 1.12
Suprachiasmatic nu	3.55 ± 0.90	3.73 ± 1.18
Globus pallidus	3.28 ± 0.71	3.83 ± 1.38
Bed nu striaterminalis	3.44 ± 0.76	3.58 ± 1.15
Olfactory tubercle	4.41 ± 1.06	4.39 ± 1.34
Diagonal band		
Dorsal	4.36 ± 1.07	3.92 ± 1.31
Ventral	4.11 ± 1.05	3.80 ± 1.36
Amygdala basolat/med	3.95 ± 0.86	3.65 ± 0.96
Hippocampus		
CA1	3.91 ± 0.85	3.36 ± 0.77
CA2	4.00 ± 0.91	3.41 ± 0.80
CA3	4.11 ± 0.92	3.69 ± 0.86
Dentate gyrus	4.29 ± 0.99	4.18 ± 1.05
SLM	4.49 ± 1.01	4.91 ± 1.92
Accumbens nucleus	3.45 ± 0.83	3.33 ± 1.16
Caudate putamen		
Dorsal	4.33 ± 1.00	3.67 ± 1.40
Ventral	4.35 ± 1.09	3.80 ± 1.29
Lateral	4.39 ± 1.00	3.76 ± 1.34
Medial	4.36 ± 1.04	3.68 ± 1.32
Septal nucleus lateral	3.90 ± 0.88	3.31 ± 1.20
medial	4.19 ± 1.01	3.84 ± 1.15
***Diencephalon***		
Habenular nu lateral	5.50 ± 1.15	5.75 ± 1.85
Habenular nu medial	5.48 ± 1.67	5.12 ± 1.60
Lateral geniculate nu dorsal	4.61 ± 1.91	4.19 ± 1.16
Medial geniculate nu	4.59 ± 1.81	4.83 ± 1.61
Thalamus		
Ventroposterior lateral nu	4.78 ± 2.14	4.08 ± 0.96
Ventroposterior medial nu	4.99 ± 2.15	4.31 ± 1.06
Paratenial nu	4.61 ± 0.88	4.46 ± 1.76
Anteroventral nu	5.91 ± 1.26	5.84 ± 2.41
Anteromedial nu	4.95 ± 1.11	4.61 ± 1.77
Reticular nu	4.98 ± 1.09	4.93 ± 2.08
Paraventricular nu	4.41 ± 0.87	4.23 ± 1.68
Parafascicular nu	4.88 ± 1.31	4.85 ± 1.70
Subthalamic nucleus	4.77 ± 1.47	4.93 ± 1.71
Hypothalamus		
Supraoptic nu	5.09 ± 1.47	5.34 ± 1.41
Lateral	3.89 ± 0.93	3.75 ± 1.29
Anterior	3.91 ± 1.00	3.90 ± 1.25
Periventricular	3.40 ± 1.00	3.29 ± 1.05
Arcuate	4.32 ± 1.20	4.66 ± 1.79
Ventromedial	4.03 ± 1.13	4.54 ± 1.87
Posterior	4.52 ± 0.92	4.01 ± 1.43
Mammillary nucleus	3.99 ± 1.62	4.59 ± 1.48
Zone Incerta	4.36 ± 1.40	4.18 ± 1.27
***Mesencephalon***		
Interpeduncular nucleus	5.09 ± 1.42	5.89 ± 2.18
Substantianigra	3.50 ± 0.99	4.45 ± 1.75
Pretectal area	4.07 ± 1.18	4.92 ± 1.92
Superior colliculus	4.17 ± 1.07	5.38 ± 2.28
Deep layers	4.05 ± 1.25	4.71 ± 1.12
Inferior colliculus	6.62 ± 2.06	7.01 ± 2.52
***Rhombencephalon***		
Flocculus	5.67 ± 1.68	6.16 ± 2.24
Cerebellar gray matter	5.58 ± 1.65	6.59 ± 2.31
Molecular layer cerebellar		
gray matter	6.54 ± 1.45	7.23 ± 2.96
***White matter***		
Corpus callosum	2.74 ± 0.68	2.74 ± 1.08
Internal Capsule	3.19 ± 0.78	3.30 ± 1.02
Cerebellar white matter	4.56 ± 1.67	5.38 ± 1.83
***Non-blood–brain barrier regions***		
Subfornical organ	4.02 ± 0.93	4.72 ± 3.10
Median eminence	5.03 ± 1.87	5.44 ± 1.73
Choroid plexus	15.89 ± 6.07	16.66 ± 6.90

k* = (ml/s/g) x 10^−4^. Values are mean ± S.D of n = 6 saline and 7 quinpirole. Each region of interest was measured in sextuplicate in each rat.

### Incorporation rate (J_in_)

Regional rates of unesterified DHA incorporation into the brain, calculated from the product of k* and unesterified plasma DHA concentration (2), did not differ significantly between the groups in the 81 regions examined (data not shown). Mean J_in_ values in vehicle and quinpirole treated rats were 58.8 ± 20.8 and 73.6 ± 22.5 nmol/s/g×10^−4^, respectively (P >0.05 by unpaired t-test).

## Discussion and conclusions

D2-like receptor activation by acute quinpirole did not significantly change brain DHA incorporation coefficient (k*) or rate (J_in_) in any of the 81 regions studied. The incorporation coefficient k* reflects the metabolic loss of DHA by the brain following its release from membrane phospholipid and J_in_ reflects net loss [35]. The lack of a quinpirole effect on each parameter suggests that D2-like receptor activation does not involve DHA release from membrane phospholipid.

DHA release is controlled by iPLA_2_ [3], which is activated following displacement of bound calmodulin protein by calcium influx factor [23]. Intracellular calcium increases concurrently because it is released with the calcium influx factor from the ER/SR in response to IP_3_ formation following PLC stimulation [19–21]. Unlike iPLA_2_, cPLA_2_ requires extracellular calcium for activation [3], consistent with in vivo evidence of increased AA but not DHA incorporation into the brain following glutamatergic NMDA receptor activation, which allows extracellular calcium into the cell [16]. D2-like receptor stimulation also allows extracellular calcium into the cell, similar to NMDA [28]. Thus, the increase in AA [12, 38] but not DHA incorporation (Table 2) following acute quinpirole confirms the independence of iPLA_2_ of extracellular calcium and likely reflects selective coupling of D2-like receptors to cPLA_2_ but not iPLA_2_. Several in vitro studies reported a reduction in intracellular calcium levels following dopaminergic activation [27, 28]. The lack of effect of quinpirole on k* agrees with these observations.

This study does not rule out the possibility that intracellular calcium levels increased following D2-receptor stimulation with quinpirole, as reported in vitro [25–28]. Such elevations if present, however, were not significant enough to produce a measurable DHA signal with our method.

This study demonstrates that there is no DHA signal following activation of D2-like receptors, although there is a signal following activation of muscarinic and serotonergic receptors [9, 17]. It is likely, therefore, that iPLA_2_ is activated by the latter two but not D2 receptors. The difference in G-protein receptor coupling remains to be clarified, but it may be related to limited intracellular calcium release and dissociation of calmodulin from iPLA_2_ following dopaminergic receptor stimulation, compared to muscarinic stimulation [39–42].

In summary, this study showed that D2-like receptor activation does not involve DHA release as a second messenger. This observation, along with our previous finding that D2-like receptor activation stimulates AA release, suggest that D2 receptors are selectively coupled to AA but not DHA as a second messenger.

## Electronic supplementary material

Additional file 1: Table S1.: Plasma unesterified fatty acid concentrations (nmol/ml) in rats treated with saline or quinpirole. (DOC 40 KB)

## Abbreviations

AA: Arachidonic acid
cPLA_2_: Ca^2+^-dependent cytosolic phospholipase A_2_
iPLA_2_: Ca^2+^-independent phospholipase A_2_
DHA: Docosahexaenoic acid
ER/SR: Endoplasmic/sarcoplasmic reticulum (ER/SR)
IP_3_: inositol 1,4,5-triphosphate
PIP_2_: phosphatidylinositol 4,5-bisphosphate
PLC: Phospholipase C
PUFA: Polyunsaturated fatty acids
TLC: Thin layer chromatography.
