# Supplementary material for: D2-like receptor activation does not initiate a brain docosahexaenoic acid signal in unanesthetized rats
Source: BMC Neurosci. 2014 Oct 30;15:113. doi: 10.1186/1471-2202-15-113 (PMC4295280; doi:10.1186/1471-2202-15-113)
Supplement: Supplementary file 1 — Additional file 1: Table S1.: Plasma unesterified fatty acid concentrations (nmol/ml) in rats treated with saline or quinpirole. (DOC 40 KB) [file 12868_2014_3806_MOESM1_ESM.doc]

# Table S1: Plasma unesterified fatty acid concentrations (nmol/ml) in rats treated with saline or quinpirole

|  | Saline (n=7) | Quinpirole (n=8) |
| --- | --- | --- |
| Palmitic (16:0) | 137± 34 | 142± 46 |
| Palmitoleate (16:1n-7) | 16 ± 7 | 14 ± 5 |
| Stearic (18:0) | 38 ± 7 | 44 ± 17 |
| Oleic (18:1n-9) | 79 ± 24 | 83 ± 26 |
| Vaccenic (18:1n-7) | 15 ± 3 | 16 ± 5 |
| Linoleic (18:2n-6) | 100 ± 24 | 128 ± 35 |
| α-linolenic (18:3n-3) | 8 ± 2 | 9 ± 3 |
| Arachidonic (20:4n-6) | 10 ± 1 | 12 ± 3 |
| Eicosapentaenoic (20:5n-3) | 5 ± 1 | 6 ± 1 |
| Docosatreinoic (22:4n-6) | 21 ± 4 | 22 ± 10 |
| n-6 docosapentaenoic (22:5n-6)^1^ | 0.4 ± 0.1 | 0.4 ± 0.2 |
| n-3 docosapentaenoic (22:5n-3) | 6 ± 1 | 8 ± 1 |
| Docosahexaenoic (22:6n-3) | 13 ± 2 | 17 ± 4 |
| Total saturated fatty acids | 175 ± 40 | 186 ± 62 |
| Total monounsaturated fatty acids | 110 ± 33 | 112 ± 36 |
| Total n-6 polyunsaturated fatty acids | 131 ± 26 | 162 ± 43 |
| Total n-3 polyunsaturated fatty acids | 28 ± 5 | 33 ± 8 |
| Total fatty acids | 448 ± 102 | 500 ± 147 |

Data are mean ± SD of n = 6 saline and 7 quinpirole treated rats. ^1^Mean of 5 per group for n-6 docosapentaenoic acid because it was not detected in 1 control and 2 quinpirole rats.
